# Phylogenetic Analyses of Lizards from the Chilean Humboldt Archipelago Reveal a New Species for the Chañaral Island (Squamata: Liolaemidae) †

**DOI:** 10.3390/ani13223576

**Published:** 2023-11-19

**Authors:** Ricardo Campos-Soto, Evelyn Rodríguez-Valenzuela, Yareta Bruna, Gabriel Díaz-Campusano, Franco Cianferoni, Dusan Boric-Bargetto, Fernando Torres-Pérez

**Affiliations:** 1 Escuela de Ciencias Agrícolas y Veterinarias, Universidad Viña del Mar, Viña del Mar 2572007, Chile; 2 Instituto de Biología, Facultad de Ciencias, Pontificia Universidad Católica de Valparaíso, Valparaíso 2373223, Chile; evelyn.rodriguez.v@pucv.cl (E.R.-V.); yareta.bruna@gmail.com (Y.B.); gabrieldiazcampusano@gmail.com (G.D.-C.); franco.cianferoni.a@gmail.com (F.C.); dusan.boric@pucv.cl (D.B.-B.); fernando.torres@pucv.cl (F.T.-P.)

**Keywords:** new species of *Liolaemus*, island lizards, Chañaral Island lizards, humboldt penguin national reserve, *zapallarensis* group, *Liolaemus carezzae* sp. nov.

## Abstract

**Simple Summary:**

Within the genus *Liolaemus*, the *zapallarensis* group is restricted to semi-arid and arid coastal habitats of the Atacama Desert in north-central Chile. While lizards of the *zapallarensis* group inhabit various islands of the Humboldt Archipelago, knowledge regarding the specific identification of their species is limited. To address this gap, we conducted phylogenetic analyses and examined morphological characteristics to shed light on the lizard species inhabiting these islands. Our findings reveal that lizards from the Damas, Choros, and Gaviota islands belong to *Liolaemus silvai*. In contrast, the lizards on Chañaral Island formed a distinct and previously unknown species and were clearly distinctive from *Liolaemus silvai*. In light of our phylogenetic and morphological results, we postulate that the lizards inhabiting Chañaral Island constitute a novel species which we have described as *Liolaemus carezzae* sp. nov. This study offers valuable insights into the unique endemic biodiversity found in Chile.

**Abstract:**

The Humboldt Archipelago, situated on Chile’s north-central coast, is renowned for its exceptional biodiversity. However, lizards of the *Liolaemus* genus are a particularly understudied group in this archipelago. *Liolaemus* genus is divided into two clades: *chiliensis* and *nigromaculatus*. Within the *nigromaculatus* clade the *zapallarensis* group is restricted to the semi-arid and arid coastal habitats of the Atacama Desert in north-central Chile. While it has been reported that lizards from the *zapallarensis* group inhabit various islands within the Humboldt Archipelago, there has been limited knowledge regarding their specific species identification. To identify the lizard species inhabiting these islands, we conducted phylogenetic analyses using a mitochondrial gene and examined morphological characteristics. Our findings reveal that lizards from the Damas, Choros, and Gaviota islands belong to *Liolaemus silvai*. In contrast, the lizards on Chañaral Island form a distinct and previously unrecognised group, clearly distinguishable from *Liolaemus silvai*. In conclusion, our study not only confirms the presence of *L. silvai* on the Damas, Choros, and Gaviota islands but also describes a new lizard species on Chañaral Island named *Liolaemus carezzae* sp. nov. These findings contribute valuable insights into the biodiversity of these islands and introduce a newly discovered endemic taxon to the region, enriching our understanding of Chile’s unique island ecosystems.

## 1. Introduction

The Humboldt Archipelago, located along the north-central coast of Chile is considered a biodiversity hotspot, bearing significant ecological importance [1,2,3]. Consisting of eight islands, this archipelago provides protected refuge for just three of them: Damas, Choros, and Chañaral, which collectively constitute the Humboldt Penguin National Reserve [3,4].

Within the Humboldt archipelago, the lizards belonging to the *Liolaemus* genus emerge as a particularly understudied group, despite being one of the most species-rich taxa in the Americas [5]. This taxonomically rich genus comprises nearly 270 distinct species, primarily distributed within the southern region of South America. The origins and adaptive radiation of *Liolaemus* lizards have been linked to the Andes mountain range, with this remarkable process spanning a period of at least ten to twelve million years [5,6]. Numerous instances of migration to varying latitudinal and altitudinal environments have actively promoted active cladogenesis within this genus, maintaining a fairly consistent evolutionary rate throughout its phylogenetic history [7]. The rapid emergence of diverse and fragmented geological systems, such as oceanic islands and mountain ranges, has offered many opportunities for some groups to undergo explosive radiations, particularly because these systems provide unoccupied niches free of competing species, offering new spaces for adaptation [5,8]. Another factor well-documented that promoted diversification of *Liolaemus* lizards was the climate changes during the Pleistocene due to distributional changes of their populations caused by temperature variations [9].

*Liolaemus* species have colonised nearly every available habitat in central southern and southern South America [5]. This wide geographical and environmental distribution within the *Liolaemidae* family has contributed to the evolution of an extraordinary diversity of morphologies, ecologies, and patterns of sexual behaviour, as well as variations in parity mode such as viviparous and oviparous characteristics in *Liolaemus* [5,10]. Notably, *Liolaemus* exhibits a remarkable degree of endemism, with several species being restricted to their specific type locality [11].

*Liolaemus* is taxonomically divided into two monophyletic clades, known as subgenera, named *Eulaemus* and *Liolaemus* (*sensu stricto*) [5,7,12]. *Liolaemus* (*sensu stricto*) comprises approximately 110 species, predominantly distributed in Chile and Argentina. Within this subgenus, two distinct clades, the *chiliensis* and *nigromaculatus,* have been identified. Within the *nigromaculatus* clade, we find the *zapallarensis* group, which is endemic to Chile. Its distribution encompasses Mediterranean and semi-arid regions in central Chile, as well as arid areas of the Atacama Desert, particularly in coastal habitats [5,13,14].

The *zapallarensis* group comprises five recognised species: *L. atacamensis*, *L. nigromaculatus*, *L. silvai*, *L. melaniceps*, and *L. zapallarensis* [14]. It has been reported that the *Liolaemus* species belonging to the *zapallarensis* group inhabit various islands within the Humboldt archipelago. Notably, *L. melaniceps* exhibits a restricted distribution solely on Chungungo island [13,15], a finding supported by phylogenetic analysis [14]. On Damas Island, earlier records had initially identified the presence of *L. zapallarensis* individuals [15]. However, subsequent molecular phylogenetic analyses led to their reclassification as an insular population of *L. silvai* [14]. It is important to emphasise that, up to the present time, only the Chungungo and Damas Islands have been subject to molecular investigations concerning *Liolaemus* lizards within the Humboldt archipelago [14]. Consequently, the species identity of *Liolaemus* populations inhabiting the Gaviota, Choros, and Chañaral Islands remains unresolved.

In this study, mitochondrial gene sequences were employed to uncover the phylogenetic relationships and lineages of the lizards inhabiting the Choros, Chañaral, Damas, and Gaviota islands. Complementing the molecular data, we also incorporated morphological characteristics and insights from the natural history of these lesser-known lizards. In light of phylogenetic analyses, morphological characterisation, and geographic isolation, we postulate that the lizards inhabiting Chañaral Island constitute a novel species within the *zapallarensis* group. Consequently, we provide a detailed taxonomic description of this newfound taxon. The valuable findings of this study offer a better understanding of island biodiversity, notably marked by the revelation of a new endemic lizard species in Chile.

## 2. Materials and Methods

### 2.1. Areas of Lizard Collections

**Figure 1:**
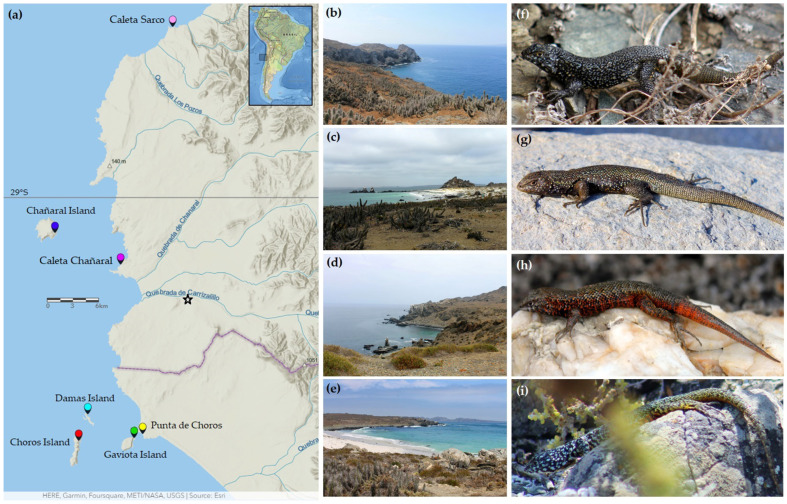
(**a**) Map of the sampled localities. Star indicates the type locality of *Liolaemus silvai*. (**b**) Landscape of Chañaral Island. (**c**) Landscape of Damas Island. (**d**) Landscape of Choros Island. (**e**) Landscape of Gaviota Island. (**f**) Lizard from Chañaral Island *Liolaemus carezzae* sp. nov. CFFPUCV-1119. (**g**) Lizard from Damas Island *L. silvai*. (**h**) Lizard from Choros Island *L. silvai*. (**i**) Lizard from Gaviota Island *L. silvai*. Photographs (**b**–**e**,**g**,**h**) taken by Ricardo Campos-Soto. Photographs (**f**,**i**) taken by Marisol Arriagada.

Lizard sampling was conducted during the summer of 2019, encompassing four coastal islands located in northern Chile. Specifically, we collected specimens from Chañaral Island (situated approximately 8.5 km away from the mainland in the Atacama Region), Damas Island (positioned at 5.5 km from the mainland), Choros Island (located approximately 8.6 km from the mainland), and Gaviota Island in the Coquimbo Region (situated at 0.5 km from the mainland). Within the mainland regions, we collected specimens of *L. silvai* from Punta de Choros in the Coquimbo Region and Caleta Chañaral in the Atacama Region (Figure 1). Additionally, individuals of *L. zapallarensis* from Los Molles in the Valparaíso Region were included in the phylogenetic analyses. Detailed information regarding the number of collected individuals and their respective geographical coordinates can be found in Table 1. The map illustrating the sampled localities was created using the ArcGIS Online platform [16].

### 2.2. Lizard Sampling

Lizards were manually captured using a rope equipped with a slipknot, and each specimen was subsequently photographed. Additionally, a tissue sample was meticulously collected from the tip of the tail for further analysis. Upon capture, the tissue samples were placed in Eppendorf tubes containing 70% ethanol. Subsequently, these individuals were released back at their respective capture points. However, four adults, comprising three males and one female from Chañaral Island, as well as one male of *L. silvai* from Punta de Choros, were humanely euthanised using an isoflurane overdose. These specimens were then preserved in 70% ethanol and deposited in the Colección de Flora y Fauna at the Pontificia Universidad Católica de Valparaíso (CFFPUCV). All procedures strictly adhered to the protocols recommended by the American Society of Ichthyologists and Herpetologists [17].

### 2.3. Obtaining Mitochondrial DNA Sequences

DNA was isolated from tail samples employing the DNeasy^®^ Blood & Tissue kit by QIAGEN (Hilden, Germany). The procedure was executed in accordance with the manufacturer’s guidelines. Subsequently, the DNA was subjected to two elution steps, with 100 μL of elution buffer employed on each occasion. For each sample, a PCR amplification was conducted targeting a 1038 bp segment of the NADH dehydrogenase subunit 2 (*ND2*) using a thermocycler Bioer model TC-96/G/H(b)C LifeEco^®^ (Hangzhou, China) and the polymerase SapphireAmp^®^ fast PCR Master Mix (Takara, Kusatsu, Japan). The primers used for amplification were as follows: forward primer 5′-CCCCGAAAATGTTGGTTTAA-3′ and reverse primer 5′-TCTGAGTTGCATTCAGGAGA-3. These primers were designed based on the alignment of *Liolaemus* sequences available in GenBank (KJ452294–KJ452325) using the software Bioedit 7.0.4.1 [18]. The cycling parameters were as follows: 30 s at 94 °C, followed by 40 cycles at 94 °C for 30 s, 55 °C for 30 s, and 72 °C for 1 min.

To confirm the efficacy of the amplification process, electrophoresis was performed on a 1% agarose gel. Following this, Macrogen Inc. (Seoul, Republic of Korea) conducted bidirectional DNA strand sequencing employing the same PCR primers. After acquiring the sequencing data, the sequences were edited to generate consensus sequences utilising Bioedit 7.0.4.1 [18]. Subsequently, alignment was carried out using Clustal W [19], an integrated feature within Bioedit v7.2.5. Further validation was performed by visually inspecting the raw chromatogram data for each individual, with a focus on sites displaying nucleotide substitutions. *ND2* gene sequences were deposited in GenBank with accession numbers OQ625482–OQ625500 and OQ632905–OQ632907 (Table S1).

### 2.4. Phylogenetic Relationships and Divergence Dates Analyses

Haplotypes were obtained from the sequences using DnaSP6 [20]. Phylogenetic analyses were performed using haplotype sequences and also incorporated sequences from species currently classified within the *zapallarensis* group which were available in GenBank [13] (Table S1). *L. platei* was used as an outgroup based on their phylogenetic proximity to the *zapallarensis* group [5,13,14]. The nucleotide substitution model was chosen via Bayesian Information Criterion (BIC) using Smart Model Selection [21] in the ATGC bioinformatics platform [22]. The selected model was identified as HKY + G. Phylogenetic reconstruction was inferred using the Bayesian Markov Chain Monte Carlo (BMCMC) method, implemented in BEAST v.2.6.5 [23]. The *ND2* matrix was partitioned by codon position, and tree linkage was accomplished using BEAUti within BEAST v.2.6.4. A Markov Chain Monte Carlo (MCMC) analysis was conducted with 5 × 10^7^ generations and sampling every 5000 generations using BEAST v.2.6.5. In this analysis, a Yule model was used as prior, and an uncorrelated lognormal relaxed molecular clock was employed, calibrated with a mutation rate of 0.023 substitutions/site/million years (as previously reported for cytochrome b in *Liolaemus*) [24]. This mutation rate was used as mean in a normal distribution in the prior for the molecular clock to estimate the best mutation rates for the data. A calibrated date of divergence between *L. silvai* and *L. zapallarensis* (mean 2.6 million years ago; Mya) estimated in [5] was used with the option “add prior”. The estimated mutation rate was 0.012 and using this rate and the Yule model as priors, uncorrelated lognormal relaxed and strict molecular clocks were run with the same chain length and sampling. The best molecular clock was selected using Nested Sampling package [25] available in BEAST v.2.6.5 and using 100 particles in each model run. This analysis estimates the log Marginal Likelihood (log (ML)) for each model which was used to calculate the log Bayes Factor (log (BF)). If log(BF) is larger than 1, the first model is favoured [26]. With the Yule model and the selected strict molecular clock, two separate MCMC runs of 5 × 10^7^ generations with sampling every 5000 generations were run using the BEAST v.2.6.5 program. The convergence based on the effective sample size (ESS) values of each run was verified in Tracer v.1.7 [27] and combined in LogCombiner v2.6.5. We accepted ESS values > 200; however, the ESS values obtained in each run were >5000. We discarded the initial 10% of trees for each run. The statistical uncertainty was depicted in values of the 95% Highest Probability Density (HPD) and node support was evaluated with posterior probabilities (pp); only pp > 0.95 were accepted as supported. The consensus tree was calculated with the maximum clade credibility and mean node heights option using TreeAnnotator v2.6.3. The consensus tree was visualised and edited in FigTree v1.4.4.

To illustrate the geographical distribution of haplotypes, we constructed an unrooted phylogeographic network using genetic sequences from both insular and mainland (adjacent to the island) regions. Additionally, we included available *L. silvai* sequences from GenBank (Table S1). For this analysis, we utilised the Median Joining method [28] and implemented it using PopART v1.7 software [29].

### 2.5. Morphological Characters

Morphological characters were defined based on body size measurements and scale counts [13,30,31]. Body measurements and scale counts of four individuals captured on Chañaral Island were acquired using the same metrics that were employed in the original description of *L. silvai* [13,30]. We used a KERN binocular stereomicroscope (magnification range: 7–45×), and measurements were obtained using a digital caliper with a precision of 0.1 mm. The morphological characters used were as follows: scales around the midbody (SABs), dorsal scales (DSs), ventral scales (VSs), head scales (HSs), number of scales between the last supralabial and the ear (SSUPEs), supralabial scales (SUPs), infralabial scales (INFSs), ciliary scales (SCIs), supraorbital scales (SORs), temporal scales, number of scales between the posterior margin of the eye and the middle of the ear (TEMPs), number of scales in contact with the interparietal scale (SCIPs), number of infradigital scales on the fourth finger (SNIFs), number of infradigital scales on the fourth toe (SINTs), snout-vent length (SVL), trunk length (TRL), width of body (WB), length of anterior part (LAP), length of posterior part (LPP), head length (HL), width head (WH), head height (HH), femur length (FL), foot length (FoL), tail length (TL), arm length (AL), and presence of precloacal pores. These measurements were subsequently compared to those of *L. silvai* [13,30].

In addition to body measurements, photographs of the dorsal scales were captured to describe their shape, following the protocol previously reported [13]. These photographs were then compared with the dorsal scales of an individual from *L. silvai*, specifically from Punta de Choros. Furthermore, colour variations were described in lizards from the Chañaral, Damas, and Choros islands. These variations were observed in the field and documented through photography.

In this study, we utilised previous publications on *Liolaemus* [30,32,33] as a guide for describing the new species and the holotype.

## 3. Results

### 3.1. Phylogenetic, Divergence Dates and Phylogeographic Analyses

By combining the newly generated sequences of this study with those of *L. silvai* from GenBank, we generated a matrix comprising a total of 60 sequences of 987 base pairs. This dataset produced a total of 23 unique haplotypes. Information regarding the number of haplotypes and their corresponding locality-specific details can be found in Table 1 and Table S1.

**Figure 2:**
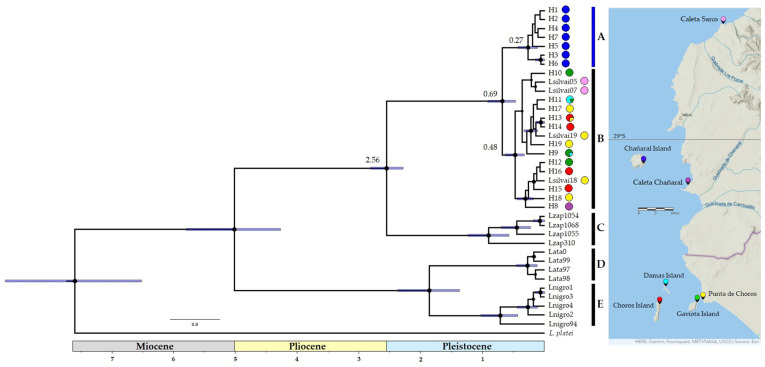
Bayesian consensus tree with divergence times based on *ND2* mtDNA gene. The values displayed at the nodes represent divergence times in millions of years, and the bars extending from the nodes indicate the 95% Highest Posterior Density (HPD) for node age. Nodes with node support exceeding 0.95 posterior probability are denoted by black circles. Circles represents the haplotypes and their colours correspond to the localities in the map. A: New clade from Chañaral Island *Liolaemus carezzae* sp. nov.; B: *L. silvai*; C: *L. zapallarensis*; D: *L. atacamensis*; E: *L. nigromaculatus*.

The phylogenetic BMCMC analyses (Figure 2) showed a noteworthy pattern where the haplotypes of lizards from the Damas, Choros, and Gaviota islands consistently clustered together within the well-supported *L. silvai* clade. The estimated divergence time for this clade was approximately 0.48 million years ago, with a credible range of 0.33 Mya to 0.64 Mya, as indicated by the highest posterior density (HPD) intervals. In contrast, lizards from Chañaral Island form a distinct, well-supported clade that is positioned as a sister group to the clade encompassing all haplotypes of *L. silvai*. The divergence time for this Chañaral Island clade is estimated at 0.27 million years ago, with an HPD interval ranging from 0.12 Mya to 0.43 Mya. Notably, the estimated divergence time between the *L. silvai* clade and the Chañaral Island clade is approximately 0.69 million years ago (HPD: 0.47–0.92). The clade consisting of *L. silvai* and Chañaral Island haplotypes was recovered as a sister group to *L. zapallarensis*.

**Figure 3:**
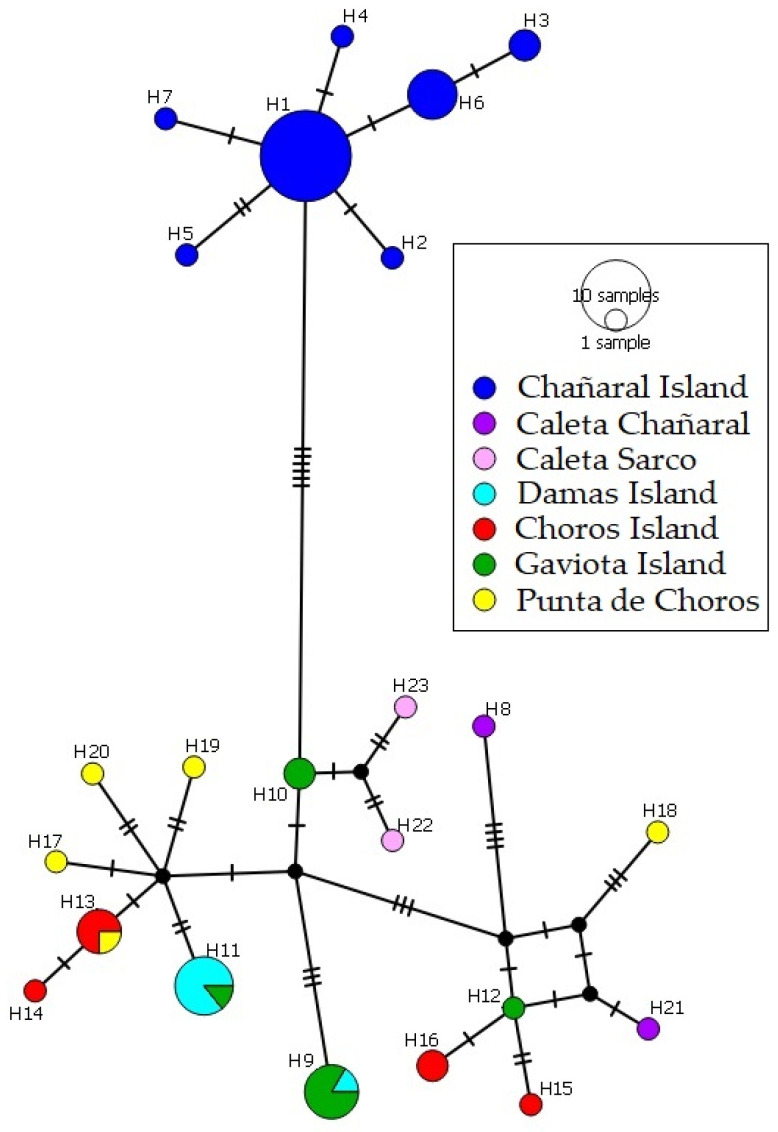
Unrooted phylogenetic network performed with *ND2* gene sequences of insular lizards and mainland *L. silvai* lizards using the Median Joining method. Colours represent the localities congruent with Figure 1 and Figure 2.

In the unrooted phylogeographic network (Figure 3), shared haplotypes were evident between the Damas and Gaviota islands, as well as between Choros Island and Punta de Choros. These findings suggest genetic affinity and potential historical gene flow between these respective island pairs. In contrast, the haplotypes originating from Chañaral Island displayed a distinct clustering pattern. Significantly, this haplogroup exhibited no shared haplotypes with any of the other sampled localities, emphasising the genetic distinctiveness of the lizard population on Chañaral Island.

### 3.2. Morphological Characteristics

Results of body measurements and scale counts are based on three adult males and one adult female captured on Chañaral Island; for males the mean is shown in parentheses. SABs = ♂ 44–49 (47), ♀ 46. DSs = ♂ 42–45 (43.3), ♀ 40. VSs = ♂ 67–71 (69.3), ♀ 69. HSs = ♂ 11 (11), ♀ 10. SSUPEs = ♂ 5–6 (5.6), ♀ 6. SUPs = ♂ 4 (4), ♀ 4. INFSs = ♂ 6 (6), ♀ 6. SCIs = ♂ 4 (4), ♀ 4. SORs = ♂ 3–4 (3.6), ♀ 4. TEMP = ♂ 8–9 (8.3), ♀ 8. SCIP = ♂ 6 (6), ♀ 6. SNIF = ♂ 10–13 (12), ♀ 16. SINT = ♂ 15–19 (16.3), ♀ 17. SVL = ♂ 61–74 (67) mm, ♀ 62 mm. TRL = ♂ 30–35 (32.3) mm, ♀ 30 mm. WB = ♂ 14–20 (16.6) mm, ♀ 14 mm. LAP = ♂ 24–27 (25) mm, ♀ 25 mm. LPP = ♂ 37–40 (39) mm, ♀ 37 mm. HL = ♂ 15–21 (17.3) mm, ♀ 15 mm. WH = ♂ 12–15 (13) mm, ♀ 11 mm. HH = ♂ 11–14 (12.3) mm, ♀ 10 mm. FL = ♂ 12–14 (13) mm, ♀ 14 mm. FoL = ♂ 20 (20) mm, ♀ 20 mm. TL = ♂ 58–88 (75.3) mm, ♀ 80 mm. AL = ♂ 21.3–21.8 (21.5) mm, ♀ 19.8 mm. Males present three precloacal pores.

Several notable differences distinguish the *Liolaemus* specimens from Chañaral Island and *L. silvai*: (i) The lizards from Chañaral Island exhibit a lower number of SAB compared to *L. silvai*, (ii) Lizards from Chañaral Island display a greater HH in comparison to *L. silvai*, (iii) The lizards from Chañaral Island possess a longer FoL than *L. silvai*, (iv) The lizards from Chañaral Island feature a reduced SINT in contrast to *L. silvai*, (v) Lizards from Chañaral Island show a greater maximum SVL (74 mm) compared to *L. silvai* (68.1 mm [30], 71.9 mm [13]). Results of the comparative analysis of body measurements between the *Liolaemus* specimens from Chañaral Island and *L. silvai* are shown in the Table S2.

**Figure 4:**
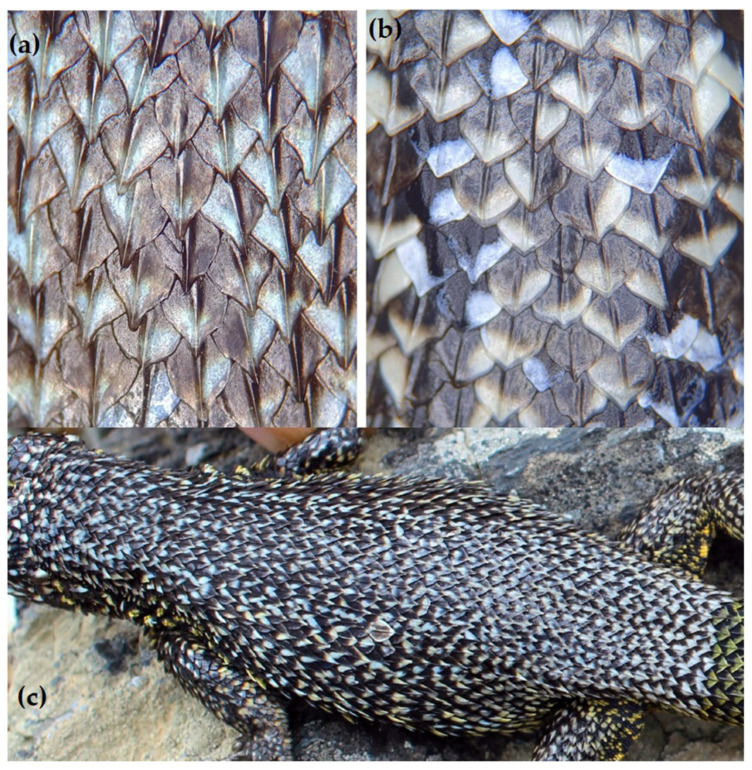
Dorsal scale comparison: (**a**) Lizard from Chañaral Island *Liolaemus carezzae* sp. nov. (**b**) *L. silvai*. (**c**) Dorsal scales naked eye of lizard from Chañaral Island *L. carezzae* sp. nov. Photographies (**a**,**b**) taken by Ricardo Campos-Soto. Photograph (**c**) taken by Marisol Arriagada.

The dorsal scales of lizards from Chañaral Island exhibit distinctive characteristics that are readily observable to the naked eye. These scales are lanceolate in shape, closely overlapping in an imbricate pattern, prominently keeled, and notably mucronate (Figure 4).

**Figure 5:**
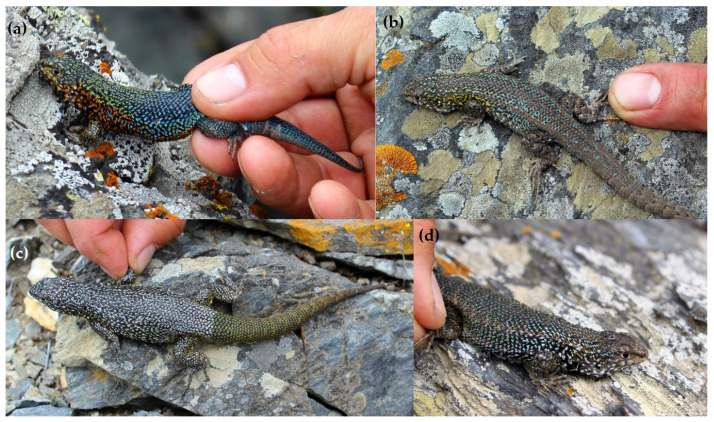
Holotype and paratypes of *Liolaemus carezzae* sp. nov. (**a**) Male holotype CFFPUCV-1117. (**b**) Female paratype CFFPUCV-1115. (**c**) Male paratype CFFPUCV-1119. (**d**) Male paratype CFFPUCV-1118. Photographs by Marisol Arriagada.

The lizards of the Chañaral, Damas, and Choros islands exhibit a remarkable array of colour variations. On Chañaral Island, male lizards were observed displaying a striking colouration pattern. The first anterior half of their bodies showcase shades of green or yellow, transitioning to a vivid blue hue in the posterior half. Their flanks display an orange colouration in the anterior third (see Figure 5a and Figure S1). In contrast, females display lighter colour variations (Figure 5b). Other male lizards from Chañaral Island exhibit a uniform melanic colouration, featuring black scales adorned with spots of yellow, green, or light blue (Figure 5c). Ventrally, the posterior abdomen display a subtle orange tint but is predominantly characterised by extensive melanic reticulation. Additionally, although not photographed, certain individuals from Chañaral Island display a distinctive burgundy colouration.

In *L. silvai* lizard from Damas Island predominantly display a uniform melanic colouration, with black scales adorned with yellow, green, or light blue spots (Figure S2a,c). On the ventral side, the posterior abdomen exhibit an orange colouration with black reticulation (Figure S2b), although this orange hue is less conspicuous in melanic individuals (Figure S2d).

In contrast, lizards from Choros Island exhibit predominantly dark or light brown colours (Figure S2e). Ventral colouring, extending from the head to the tail, display vibrant red or orange hues, particularly pronounced in the gular zone and less so in the abdomen (Figure S2f). Laterally, from the neck to the tail, an orange colouration is also evident (Figure 1h).

### 3.3. Description of the New Species

The classification of individuals from Chañaral Island within the genus *Liolaemus* relies on several morphological diagnostic characters reported by [34], and the similarity of characters reported with *L*. *silvai* [30].

#### *Liolaemus carezzae* sp. nov. CAMPOS-SOTO, CIANFERONI and TORRES-PÉREZ

**Figure 6:**
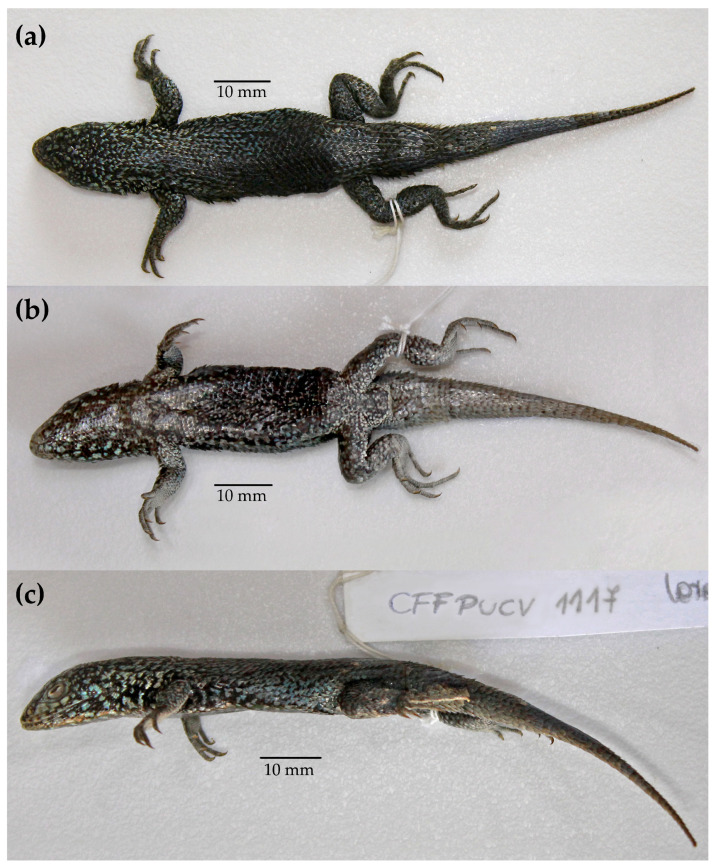
Preserved male holotype of *Liolaemus carezzae* sp. nov. CFFPUCV-1117. (**a**) Dorsal view. (**b**) Ventral view. (**c**) Lateral view. Photographies taken by Josefa Borcosque.

Holotype: Colección de Flora y Fauna Pontificia Universidad Católica de Valparaíso (CFFPUCV) 1117, adult male specimen (Figure 5a, Figure 6 and Figure S1) collected on Chañaral Island 29°2′17″ S, 71°34′2″ W, 60 m above sea level, Atacama Region, Chile. Collector Ricardo Campos-Soto, 21 January 2019.

Paratypes: CFFPUCV1118–1119 two adult male specimens (Figure 5c,d). CFFPUCV-1115 adult female (Figure 5b). All captured in the same place of the holotype. Collector Ricardo Campos-Soto, 21 January 2019.

Etymology: This species is dedicated to Carezza Botto-Mahan whose remarkable contributions to the study of natural processes, and specifically to the ecology of Chagas disease, have been outstanding. His research highlights the importance of assessing the role of lizards in the *Trypanosoma cruzi* transmission [35].

Phylogenetic evidence: The phylogenetic analyses revealed that *Liolaemus carezzae* sp. nov. forms a distinct and well-supported clade positioned as a sister taxon to *L. silvai* and belonging to the *zapallarensis* group [7] (Figure 2). *Liolaemus carezzae* sp. nov. can be distinguished from other *Liolaemus* of the *zapallarensis* group because exhibits distinct nucleotide sequences in the *ND2* gene, as outlined in haplotypes 1 to 7 (refer to Table S1 for details).

Diagnosis: *Liolaemus carezzae* sp. nov. can be distinguished from other *Liolaemus* of the *zapallarensis* group primarily through the following characteristics: (i) Dorsal Scale Morphology: The dorsal scales of *Liolaemus carezzae* sp. nov. display a unique lanceolate shape, closely overlapping in an imbricate pattern, featuring strong keels, and pronounced mucronate tips (Figure 4, Table S2). (ii) Dorsal Colouration in Males: In terms of dorsal colouration, males of *Liolaemus carezzae* sp. nov. demonstrate vibrant green or yellow hues on the anterior half of their bodies, transitioning to a striking blue colouration on the posterior half (Figure 5a and Figure S2). (iii) Scales count and body measurements: In comparison with the measurements describes in *L. silvai* [13,30], *Liolaemus carezzae* sp. nov. exhibits several distinguishing characteristics. *Liolaemus carezzae* sp. nov. show a smaller number of SAB (mean of 3 males = 47, 1 female = 46) compared to *L. silvai* (mean of 8 males = 50, mean of 6 females = 50). *Liolaemus carezzae* sp. nov. show a greater HH (mean of 3 males = 12.3 mm, 1 female = 11 mm) in comparison to *L. silvai* (mean of 8 males = 8.6 mm, mean of 6 females = 7.6 mm). The males of *Liolaemus carezzae* sp. nov. show a longer FoL (mean of 3 males = 20 mm, 1 female = 20) compared to *L. silvai* males (mean of 8 males = 16.9 mm, mean of 6 females = 20 mm), although in [13] a similar mean in males (19.9) but lower in females (16.4) has been reported. *Liolaemus carezzae* sp. nov. show fewer SINT (mean of 3 males = 16.3, 1 female = 17) compared to *L. silvai* (mean of 8 males = 24, mean of 6 females = 23). *Liolaemus carezzae* sp. nov. show a greater maximum SVL (74 mm) in comparison to *L. silvai* (68.1 mm [30], 71.9 mm [13]). For details see Table S2.

In contrast to *L. melaniceps*, *Liolaemus carezzae* sp. nov. also exhibits noticeable differences. *Liolaemus carezzae* sp. nov. show a reduced number of SAB (among 44 to 49) compared with *L. melaniceps* (among 50 to 52) [10]. *Liolaemus carezzae* sp. nov. show a lower count of SINT (among 15 to 19) compared with *L. melaniceps* (among 25 to 27) [10]. The melanic form of *L. carezzae* sp. nov. has black scales with yellow, green or light blue spots, whereas *L. melaniceps* lacks these pots on its scales. These distinct morphological features contribute to the differentiation and identification of *Liolaemus carezzae* sp. nov. from its closely related species.

Description of Holotype: Adult male, nucleotide sequence of the *ND2* gene: haplotype 1 (accession number OQ625482). SAB = 44. DS = 43. VS = 70. HS = 11. SSUPE = 6. SUP = 4. INFS = 6. SCI = 4. SOR = 4. TEMPs = 8. SCIPs = 6. SNIFs = 13. SINTs = 15. SVL = 61 mm. TRL = 30 mm. WB = 14 mm. LAP = 24 mm. LPP = 37 mm. HL = 15 mm. WH = 12 mm. HH = 11 mm. FL = 12 mm. FoL = 20 mm. TL = 58 mm. Arm length = 21.4 mm. Tibial length = 9.2 mm. Dorsal head scales are convex. Rostral scale is rectangular with rounded edges (2.8 × 1.0 mm). Two postrostral scales with a semi-circle shape. Nasal scales with ellipsoidal shape, separate from the rostral scale and surrounded by six scales. Nostrils are circular and occupy more than half of the nasal scale. Four internasal scales in contact with supra rostral scale, the laterals are rectangular (0.5 × 08 mm.) while the medial are almost quadrangular (0.7 × 1.2 mm). Five frontonasal scales, three centrals and two laterals, the laterals have an elongated lanceolate shape while the three centrals are quadrangular with one central largest. Seven prefrontal scales, first row with a small rhomboidal medial scale and two symmetric extended mediolateral scales, second row presents two symmetric central scales and two smaller lateral scales. Frontal scale with rhomboidal shape (1.5 × 2.1 mm). Two symmetric frontoparietal scales with pentagonal shape. Interparietal scale presents a pentagonal shape. Two large parietal scales. Supercilliaries scales 9–9. Subocular scale wider than longer (4.3 × 0.9 mm). Mental with a semi-circle shape (2.6 × 1.2 mm) and surrounded by four scales. Chinshield scales 5–5. Gular and ventral body scales imbricates and rounded. Auditory meatus higher than wide (1.0 × 2.1 mm). The shape of dorsal scales is lanceolate, imbricate, strongly keeled and strongly mucronate (Figure 4a). Antehumeral spot poorly visible and irregular. Three precloacal pores. Infradigital scales from fingers are imbricates and keeled (3 keels), numbering: I: 10, II: 19, III: 17, IV: 13, V: 8. Infradigital scales from toes are imbricates and keeled (3 keels), numbering: I: 14, II: 25, III: 24, IV: 15, V: 11.

Colour in life of the holotype: The dorsal part of the head displays a combination of black colouration with green and yellow dots. The temporal zone, located on the sides of the head, features shades of orange and some blue dots. Dorsally, the first half of the body is predominantly green, transitioning into a vibrant blue colouration in the posterior half and the tail is blue. The flanks exhibit an orange hue, primarily in the anterior third of the body (Figure 5a and Figure S1). Ventrally, from the gular zone to the back of the abdomen, the colouration is predominantly black, interspersed with patches of orange and light blue dots. The pelvic zone, located on the lower part of the abdomen, displays an orange colouration, while the tail is light blue.

Colour pattern preservative: Dorsal and laterally the head and trunk display a black colouration with scales with spots of light blue hues. Dorsally, the tail is dark without appreciated scales with spots of light blue. Ventrally, from the gular zone to the back of the abdomen, the colouration is predominantly black, with light blue and whitish dots. The pelvic zone and the tail display a most whitish colouration. Green, orange, and yellow spots in the head, gular zone, and flank are absent (Figure 6).

Variation: The following meristic and morphometric values are based on two adult males and one female paratypes; for males, the mean is shown in parentheses. SABs = ♂ 48–49 (48.5), ♀ 46. DSs = ♂ 42–45 (43.5), ♀ 40. VSs = ♂ 67–71 (69), ♀ 69. HSs = ♂ 11 (11), ♀ 10. SSUPEs = ♂ 5–6 (5.5), ♀ 6. SUPs = ♂ 4 (4), ♀ 4. INFSs = ♂ 6 (6), ♀ 6. SCIs = ♂ 4 (4), ♀ 4. SORs = ♂ 3–4 (3.5), ♀ 4. TEMPs = ♂ 8–9 (8.5), ♀ 8. SCIPs = ♂ 6 (6), ♀ 6. SNIFs = ♂ 10–13 (11.5), ♀ 16. SINTs = ♂ 15–19 (17), ♀ 17. SVL = ♂ 61–74 (67.5) mm, ♀ 62 mm. TRL = ♂ 32–35 (33.5) mm, ♀ 30 mm. WB = ♂ 16–20 (18) mm, ♀ 14 mm. LAP = ♂ 24–27 (25.5) mm, ♀ 25 mm. LPP = ♂ 40 (40) mm, ♀ 37 mm. HL = ♂ 16–21 (18.5) mm, ♀ 15 mm. WH = ♂ 12–15 (13.5) mm, ♀ 11 mm. HH = ♂ 12–14 (13) mm, ♀ 10 mm. FL = ♂ 13–14 (13.5) mm, ♀ 14 mm. FoL = ♂ 20 (20) mm, ♀ 20 mm. TL = ♂ 80–88 (84) mm, ♀ 80 mm. AL = ♂ 21.3–21.8 (21.5) mm, ♀ 19.8 mm.

Colour in life variation: In general, *Liolaemus carezzae* sp. nov. exhibits a wide range of colour variations, reminiscent of the colour diversity observed in *L. silvai* as reported in previous studies [13,36]. The paratype CFFPUCV-1119 displays a colour pattern distinct from the holotype, showing a melanic colouration, characterised by black scales adorned with vibrant spots of yellow, green, and light blue hues (Figure 5c). On the other hand, the paratype CFFPUCV-1118 displays colours that are less prominent and conspicuous (Figure 5d).

Distribution and natural history: This newly described species, *Liolaemus carezzae* sp. nov., is currently known solely from its type locality on Chañaral Island, situated within the Pingüino de Humboldt National Reserve in the Atacama Region of Chile. Chañaral Island, covering an area of approximately 507 hectares, rises gently from 30 to 50 m above sea level, with its highest point reaching 150 m [37].

Chañaral Island is situated within the Coquimban Biogeographical Province [38] and is characterised by a coastal desert environment influenced by mist, creating a semi-arid climate. The island’s vegetation primarily comprises cacti, both annual and perennial herbs, and various shrub species [3].

Individuals of *Liolaemus carezzae* sp. nov. inhabit areas amongst cacti, preferably *Eulychnia acida* which provide refuge (Figure S3). Additionally, these lizards are commonly observed sheltering between cracks in rocks and beneath stones (Figure S4).

## 4. Discussion

Taxonomy plays a crucial role in understanding biodiversity, particularly on islands with unique species assemblages. Accurate identification and classification of organisms, such as lizards, provide the foundation for studying their evolutionary history, ecological interactions, and conservation needs. Exploring the biodiversity of lizards on islands contributes to our comprehension of biogeographical patterns, island colonisation processes, and the potential for endemic species, enhancing our overall understanding of island ecosystems.

Our phylogenetic analyses reveal that haplotypes of lizards from the Damas, Choros, and Gaviota islands are clustered with haplotypes of *L. silvai* in a supported clade (Figure 2). Also, the haplotypes network does not show a marked population structure between the populations of these islands (Figure 3). On the other hand, the lizard population from Chañaral Island cluster in a new supported clade and appears as a divergent group in the network which is previously unrecognised, and is clearly differentiated from the populations of the other studied islands (Figure 2 and Figure 3).

The divergence between Chañaral Island clade and *L. silvai* was estimated at ~0.7 Mya, while the most recent common ancestor for the Chañaral Island clade was estimated at ~0.27 Mya (Figure 2). Our estimations are consistent with the date of divergence estimated for scorpions of the genus *Brachistosternus* in the same island [39]. These estimates are consistent with the processes that took place during the Pleistocene Climatic Oscillations (PCOs), a frequent phenomenon in the late Pleistocene that influenced the formation of marine terraces through sea level fluctuations [40,41,42].

The variations in sea level and marine terraces affected the geographic configuration of the habitats in northern Chile, likely isolating populations on coastal islands [39,43,44]. Lizards included in the Chañaral Island clade may have originated from a population that diverged when the island emerged. Chañaral island is the highest, northernmost, and most distant island of the Humboldt Archipelago. Currently, this island has an area of 507 ha and an elevation of 30 to 50 m above sea level, reaching its highest point at 150 m [37]. It is separated from the mainland by a sea floor depth of 80 to 120 m [39]. The elevation of the island may have provided a refuge for the lizards that climbed to the summit when the sea level rose. When the sea level dropped, the lizards could have colonised the newly available habitats; however, the isolation, the distance from the mainland, and sea floor depth may have acted as barriers to gene flow, facilitating the speciation process.

The populations from the Damas, Choros, and Gaviota islands within the *L. silvai* clade have a smaller distance and sea floor depth to the mainland than the Chañaral Island population. The high sea level periods may have caused the extinction of some populations that inhabited the coastal plains, potentially leading to unoccupied habitats that could have been resettled by ancestral mainland populations during low sea level periods. That is, the shorter distance between island and mainland may have enabled a connection between populations during low sea level periods. This may account for the inclusion of the lizards from Caleta Chañaral and Caleta Sarco, in front of the Chañaral Island, in the *L. silvai* clade (Figure 2). This biogeographic scenario has also been proposed for scorpions of the genus *Brachistosternus* [39].

The lizards from Chañaral Island exhibit distinctive morphological features, such as the shape of their dorsal scales (Figure 4) and their bluish colour (Figure 5a and Figure S1), which are absent in *L. silvai* and the other members of the *zapallarensis* group. Nevertheless, we observed a conspicuous degree of chromatic variability, a phenomenon previously documented in *L. silvai* [13,36]. This colour variation may be associated with habitat characteristics, such as the presence of lichens on the rocks or the type of cacti and herbs. Although lizards from the Damas, Choros, and Gaviota islands are included in the *L. silvai* clade, they display colour differences. The most distinctive colour differences are observed between lizards from the Damas and Choros islands (Figure S2), suggesting that these insular populations could be subspecies of *L. silvai*. Additional data from other markers, such as nuclear ones, are required to assess the level of molecular divergence of these populations. On the other hand, the lizards from Gaviota Island have the most similar colouration to *L. silvai*.

Although this study did not examine the sequences of *L. melaniceps* (inhabiting on Chungungo Island), *L. carezzae* sp. nov. exhibits clear morphological differentiation from *L. melaniceps*, such as: *L*. *carezzae* sp. nov. show minor scales count around the midbody and a lower count of infradigital scales on the fourth toe. Also, the melanic form of *L. carezzae* sp. nov. has black scales with yellow, green, or light blue spots, whereas *L. melaniceps* lacks these pots on its scales [10]. These differences and the geographic isolation allow us to suggest that the lizards from Chañaral Island and the other islands do not belong to *L. melaniceps*.

The *zapallarensis* group comprises several lizard species that are endemic to the islands where they occur and have been mainly distinguished by morphologic characters and isolation. For example, in the Humboldt Archipelago, *L. ater* is restricted to Pájaros Island in front of Coquimbo, Coquimbo Region, and *L*. *melaniceps* is restricted to Chungungo Island in front of Chungungo, Coquimbo Region [13,45,46]. *L. melaniceps* has been recently validated with molecular data [14].

These findings contribute to our understanding of the biodiversity and evolutionary dynamics within this group of lizards and enrich our knowledge of the unique ecosystems in this region.

## 5. Conclusions

Our phylogenetic analyses revealed that the lizards inhabiting the Damas, Choros, and Gaviota islands can be attributed to *L. silvai*, whereas the inhabitants of Chañaral Island formed a distinct, robustly supported clade that exhibited genetic divergence from the other island populations. Considering our phylogenetic findings, coupled with morphological traits and geographical isolation, we have identified the lizards from the Chañaral Island as a novel species. We formally described this species as *Liolaemus carezzae* sp. nov., which belongs to the *zapallarensis* group. The data provided by this study offer valuable insights into the unique endemic biodiversity found in Chile.

## Figures and Tables

**Table 1:** Insular and mainland site collection of *Liolaemus* specimens used in this study.

Locality	Latitude/Longitude	Species	N	H
Chañaral Island	29°2′17″ S/71°34′8″ W	*L. carezzae* sp. nov.	28	7
Caleta Chañaral	29°4′14″ S/71°29′16″ W	*L. silvai*	1	1
Damas Island	29°13′48″ S/71°31′41″ W	*L. silvai*	7	2
Choros Island	29°15′32″ S/71°32′19″ W	*L. silvai*	7	4
Gaviota Island	29°15′24″ S/71°28′16″ W	*L. silvai*	9	4
Punta de Choros	29°15′4″ S/71°27′40″ W	*L. silvai*	4	4
Los Molles	32°14′29″ S/71°31′7″ W	*L. zapallarensis*	3	1

N: Number of individuals captured; H: number of haplotypes.

## Data Availability

The data presented in this study are available in the manuscript and in its Supplementary Material.

## Supplementary Materials

The following supporting information can be downloaded at: https://www.mdpi.com/article/10.3390/ani13223576/s1, Table S1: GenBank accession number of taxa used in this study; Table S2: Primary measurements and key characteristics exhibited by *Liolaemus carezzae* sp. nov. and *L. silvai*; Figure S1: Characteristic colouration of *Liolaemus carezzae* sp. nov.; Figure S2: Colour variation of lizards from the Damas and Choros islands; Figure S3: Male of *Liolaemus carezzae* sp. nov. taking refuge between cacti; Figure S4: Female of *Liolaemus carezzae* sp. nov. on a rocky substrate.
