# Supplementary material for: Phylogenetic Analyses of Lizards from the Chilean Humboldt Archipelago Reveal a New Species for the Chañaral Island (Squamata: Liolaemidae)
Source: Animals (Basel). 2023 Nov 19;13(22):3576. doi: 10.3390/ani13223576 (PMC10668673; doi:10.3390/ani13223576)
Supplement: Supplementary file 1 [file animals-13-03576-s001.zip › animals-2663928-supplementary.pdf]

Table S1: GenBank accession numbers of taxa used in this study

Haplotype or Taxa	*Liolaemus* species	Locality	N	Accession number
H1	*L. carezzae* sp. nov.	Chañaral Island	17	OQ625482
H2	*L. carezzae* sp. nov.	Chañaral Island	1	OQ625483
H3	*L. carezzae* sp. nov.	Chañaral Island	2	OQ625484
H4	*L. carezzae* sp. nov.	Chañaral Island	1	OQ625485
H5	*L. carezzae* sp. nov.	Chañaral Island	1	OQ625486
H6	*L. carezzae* sp. nov.	Chañaral Island	5	OQ625487
H7	*L. carezzae* sp. nov.	Chañaral Island	1	OQ625488
H8	*L. silvai*	Caleta Chañaral	1	OQ625489
H9	*L. silvai*	Gaviota (5) and Damas Island (1)	6	OQ625490
H10	*L. silvai*	Gaviota Island	2	OQ625491
H11	*L. silvai*	Damas (6) and Gaviota Island (1)	7	OQ625492
H12	*L. silvai*	Gaviota Island	1	OQ625493
H13	*L. silvai*	Choros Island, Punta Choros	4	OQ625494
H14	*L. silvai*	Choros Island	1	OQ625495
H15	*L. silvai*	Choros Island	1	OQ625496
H16	*L. silvai*	Choros Island	2	OQ625497
H17	*L. silvai*	Punta Choros	1	OQ625498
H18	*L. silvai*	Punta Choros	1	OQ625499
H19	*L. silvai*	Punta Choros	1	OQ625500
Lsilvai19	*L. silvai*	Punta Choros*	1	KJ452319*
Lsilvai18	*L. silvai*	Caleta Chañaral*	1	KJ452318*
Lsilvai07	*L. silvai*	Caleta Sarco*	1	KJ452307*
Lsilvai05	*L. silvai*	Caleta Sarco*	1	KJ452305*
Lzap310	*L. zapallarensis*	Totoralillo*	1	KJ452310*
Lzap1068	*L. zapallarensis*	Los Molles	1	OQ632905
Lzap1054	*L. zapallarensis*	Los Molles	1	OQ632906
Lzap1055	*L. zapallarensis*	Los Molles	1	OQ632907
Lnigro4	*L. nigromaculatus*	Caldera*	1	KJ452304*
Lnigro3	*L. nigromaculatus*	Caldera*	1	KJ452303*
Lnigro2	*L. nigromaculatus*	Caldera*	1	KJ452302*
Lnigro1	*L. nigromaculatus*	Caldera*	1	KJ452301*
Lnigro94	*L. nigromaculatus*	Vallenar*	1	KJ452294*
Lata0	*L. atacamensis*	El Trapiche*	1	KJ452300*
Lata99	*L. atacamensis*	El Trapiche*	1	KJ452299*
Lata98	*L. atacamensis*	Freirina*	1	KJ452298*
Lata97	*L. atacamensis*	Freirina*	1	KJ452297*
L. platei	*L. platei*	Coquimbo*	1	KJ452325*

*: Localities and accession number reported in [13]; In brackets the number of sequences of this locality when there are shared haplotypes is shown; N: number of sequences. The taxa Lsilvai19, Lsilvai18, Lsilvai07 and Lsilvai05 correspond to the haplotypes H20, H21, H22 and H23 respectively in the network (Figure 3).

Table S2. Primary measurements and key characteristics exhibited by *Liolaemus carezzae* sp. nov. and *L. silvai*. This section presents a detailed account of the primary measurements and key characteristics exhibited by individuals of *L. carezzae* sp. nov. collected from Chañaral Island, as well as individuals belonging to the species *L. silvai*.

	***Liolaemus carezzae* sp. nov.**		***L. silvai***	
	Male N=3	Female N=1	Male N=8[1]	Female N=6[1]
SAB	44-49 (47)	46	49-51 (50)[1]	49-52 (50)[1]
DS	42-45 (43.3)	40	42-45 (44)[1]	41-46 (43)[1]
VS	67-71 (69.3)	69	U[1]	U[1]
HS	11 (11)	10	11-14 (12)[1]	12-14 (13)[1]
SVL	61-74 (67)	62	55.5-68.1 (63.6)[1]	51.5-65 (57.2)[1]
TRL	30-35 (32.3)	30	28.3-33.0 (31.0)[1]	28.0-35.6 (28.8)[1]
TL	58-88 (75.3)	80	88.0-91.4 (88.7)[1]	63.8 (63.8)[1]
HL	15-21 (17.3)	15	13.7-16.5 (15.3)[1]	11.3-14.2 (12.4)[1]
HW	12-15 (13)	11	10.4-13.8 (12.4)[1]	9.4-11.5 (10.0)[1]
HH	11-14 (12.3)	10	8.0-9.5 (8.6)[1]	6.7-8.3 (7.6)[1]
FoL	20 (20)	20	14.4-18.2 (16.9)[1]	18.4-21.3 (20.0)[1]
SINT	15-19 (16.3)	17	22-26 (24)[1]	22-25 (23)[1]
AL	21.3-21.8 (21.5)	19.8	(21.6)[2]	(19.5)[2]
SDS	Lanceolate, imbricate, strongly keeled and strongly mucronate		Lanceolate or rhomboidal[2]	

SAB: Scales around the midbody. DS: Dorsal scales. VS: Ventral scales. HS: Head scales. SVL: Snout vent length. TRL: Trunk length. TL: Tail length. HL: Head length. HW: Head width. HH: Head height. FoL: Foot length. SINT: Number of infradigital scales on the fourth toe. AL: Arm length. SDS: Shape of dorsal scales N: number of individual measured. Dimension are in millimetres. In parentheses is show the mean. U: unmeasured data; [1]: Data extracted from [30]; [2]: Characteristics reported in [13].

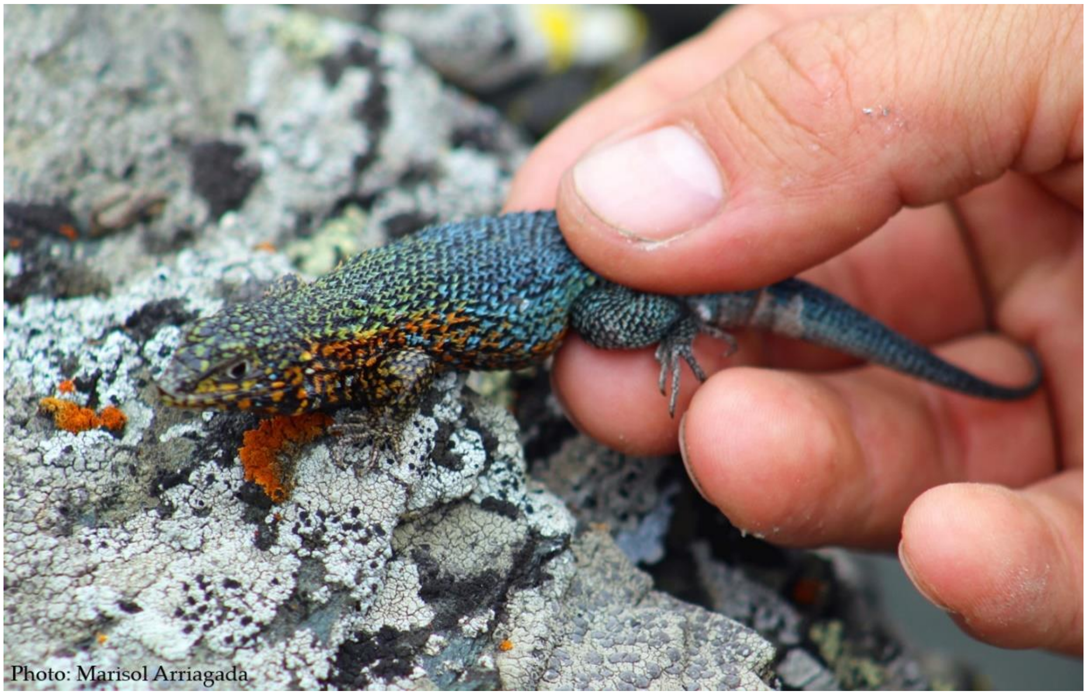

Figure S1. Characteristic colouration of *Liolaemus carezzae* sp. nov. photograph by Marisol Arriagada. Holotype CFFPUCV-1117.

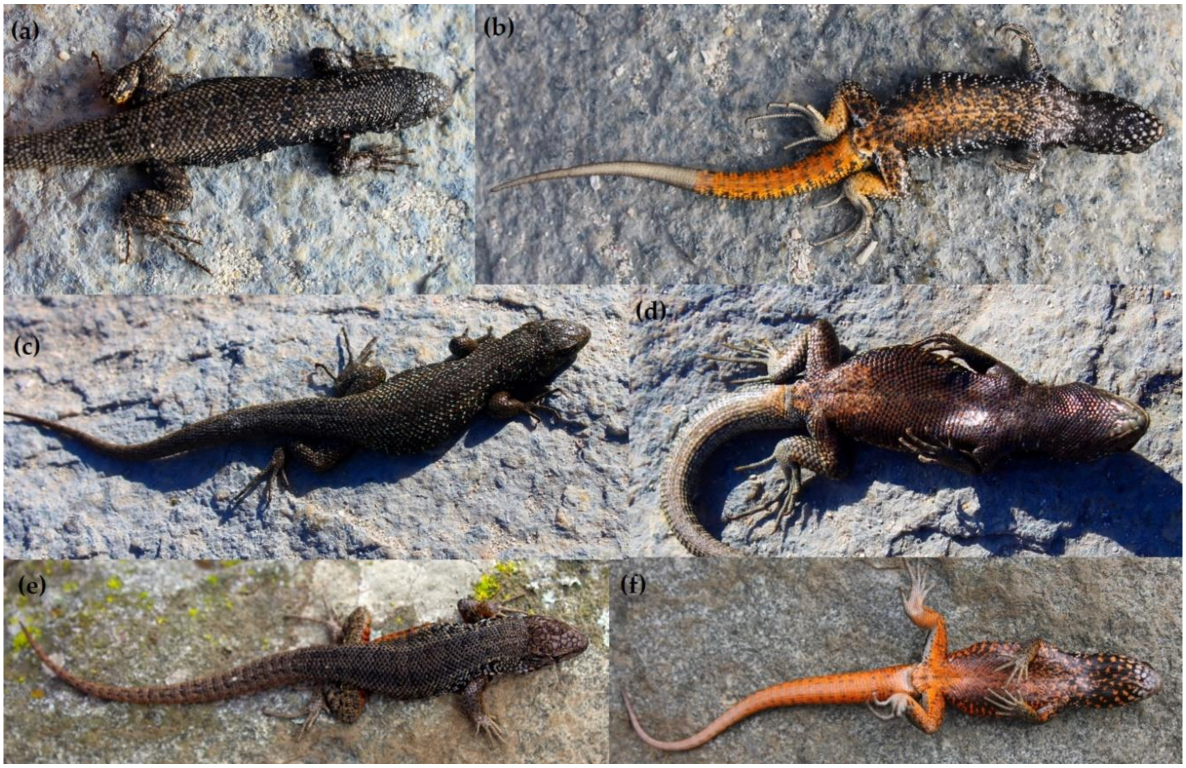

Figure S2. Colour variation of lizards from Damas and Choros Islands. (a), (b) Male from Damas Island. (c), (d) Melanic male from Damas Island. (e), (f) Male from Choros Island. Photographs by Ricardo Campos-Soto.

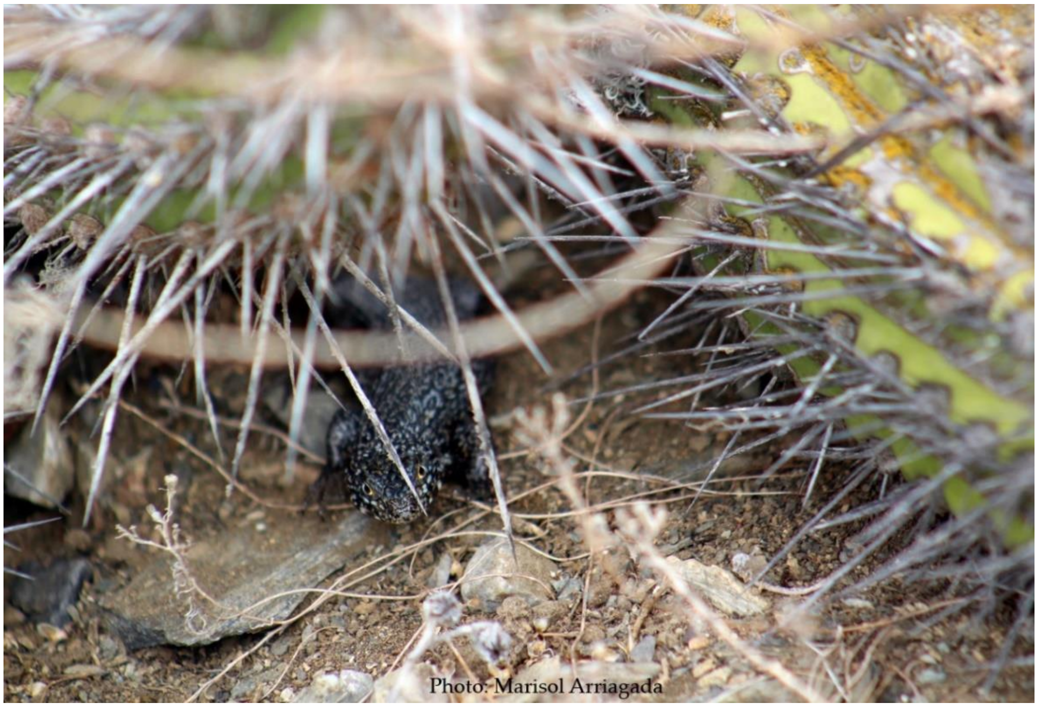

Figure S3. Male of *Liolaemus carezzae* sp. nov. taking refuge between cacti. Photograph by Marisol Arriagada. Paratype CFFPUCV-1119.

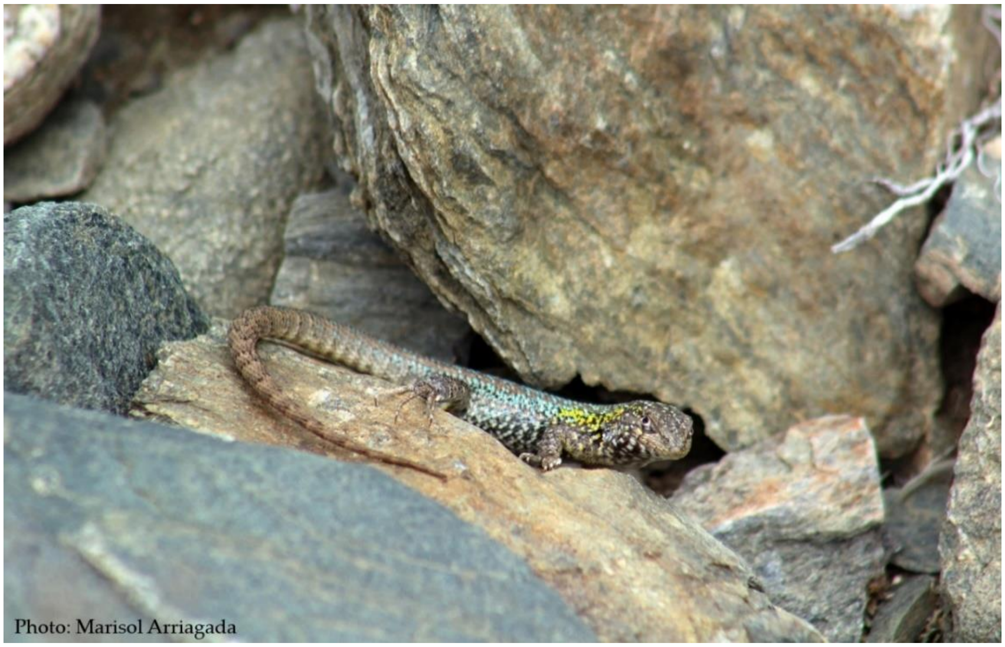

Figure S4. Female of *Liolaemus carezzae* sp. nov. on a rocky substrate. Photograph by Marisol Arriagada. Paratype CFFPUCV-1115.
